# Development and Effect of Prenatal Education Programs Using Virtual Reality for Pregnant Women Hospitalized With Preterm Labor: Experimental Study

**DOI:** 10.2196/75585

**Published:** 2025-06-30

**Authors:** SeoA Park, Hyeyoung Kim

**Affiliations:** 1 College of Nursing, Kyungwoon University, Gumi, Republic of Korea; 2 College of Nursing, Keimyung University, 1095 Dalgubeol-daero, Dalseo-Gu, Daegu, 42601, Republic of Korea, 82 01038065190

**Keywords:** nursing, pregnancy, preterm labor, prenatal care, health education, virtual reality

## Abstract

**Background:**

Pregnant women hospitalized due to preterm labor often experience anxiety, stress, and physical discomfort, which may influence uterine contractions and cervical changes, underscoring the need for effective prenatal management. Virtual reality (VR)–based prenatal education programs can enhance interaction and engagement for these women. The Cox Interaction Model of Client Health Behavior (IMCHB) provides an appropriate framework for nursing interventions addressing their complex needs. This study developed a VR prenatal education program based on the IMCHB and evaluated its effectiveness.

**Objective:**

This study aims to develop, implement, and evaluate the effects of a prenatal educational program using VR technology for pregnant women hospitalized with preterm labor.

**Methods:**

This program was developed based on the Cox IMCHB. To guide program development, we applied the Analyze, Design, Develop, Implement, and Evaluate (ADDIE) model, following its 5 stages: analysis, design, development, implementation, and evaluation. The study used a pre- and posttest design with a nonequivalent control group. A total of 15 participants in the experimental group and 16 participants in the control group, all pregnant women hospitalized with preterm labor, were analyzed. Data were analyzed using descriptive statistics; homogeneity tests; the chi-square test; the Fisher exact test; and independent, unpaired, 2-tailed *t* tests.

**Results:**

The experimental group that participated in the VR-based prenatal education program showed significantly lower levels of state anxiety (*P*=.009), stress related to preterm labor (*P*=.002), frequency of uterine contractions (*P*=.004), and intensity of uterine contractions (*P*<.001) compared with the control group. Additionally, the experimental group demonstrated a significantly greater increase in cervical length (*P*=.009). Practice behavior (*P*=.047) and self-efficacy in pregnancy health care (*P*<.001) were also significantly higher in the experimental group than in the control group.

**Conclusions:**

Prenatal education using VR was shown to be effective across physical, emotional, and educational domains by delivering a professional, integrated intervention tailored to the complex nursing needs of hospitalized patients with preterm labor.

## Introduction

### Background

Preterm labor, which involves cervical changes and uterine contractions, occurs between the 20th and 37th weeks of pregnancy and is a major cause of premature birth [1-3]. Factors associated with preterm labor are maternal age, multiple pregnancies, a history of premature birth, intrauterine infection, cervical malformation, cervical damage, abnormalities of the amniotic fluid or placenta, and fetal malformations [4,5]. Currently, the general management of pregnant women in preterm labor includes hospitalization, bed rest, and administration of painkillers to prolong the pregnancy [2]. During this process, pregnant women in preterm labor are separated from their families; must adapt to an unfamiliar hospital environment; and often experience anxiety, stress, physical discomfort, and fatigue. These are due to restrictions on activity from absolute rest, the possibility of premature birth, and concerns about the well-being of the fetus [6,7]. Pregnant women in preterm labor are known to have higher anxiety levels and face more stressors than women with normal pregnancies [8], and they often experience confusion because the exact cause of preterm labor is unknown [6]. As there is no ideal treatment beyond bed rest and analgesics after the diagnosis of preterm labor, these women experience uncertainty regarding the maintenance of pregnancy and its prognosis [9,10]. Therefore, nurses should help minimize this confusion by providing knowledge related to pregnancy and preterm labor and by offering prenatal education to reduce psychological anxiety [9]. Pregnant women who acquire prenatal knowledge can improve their self-management skills and gain confidence related to pregnancy, making prenatal education important for managing their health [11,12]. To promote physical, psychological, and physiological relaxation and deliver information to high-risk pregnant women, systematic education by experts and the use of various media are necessary [13,14]. As a method of prenatal education, relaxation therapy is often used to relieve the physical, psychological, and physiological tension associated with anxiety and stress in patients [15,16]. Among relaxation therapies, abdominal breathing is a commonly used method that can be applied in an integrated manner [17], and imagery therapy is a relaxation technique that reduces stress or anxiety by recalling visual images and expressing physical and emotional responses [15]. Hospitalized pregnant women in preterm labor experience anxiety and stress due to unexpected situations and the unfamiliar hospital environment. This leads to nervousness and triggers the release of prostaglandins through physiological mechanisms linked to psychological reactions, which, in turn, promote cervical softening and uterine contractions [5]. Therefore, applying relaxation therapy to pregnant women in preterm labor can positively affect the degree of cervical changes and contractions. Recently, the use of virtual reality (VR) technology has been expanding in the medical field, and it is emerging as a new educational method in clinical practice [18,19]. Previous studies using VR technology have reported that VR relaxation meditation programs positively affect pain control in clinical patients [20,21]. VR has also been found effective in managing anxiety and depression in patients with bipolar disorder [22], in controlling the anger among juvenile criminals through VR-based cognitive behavioral programs [23], and in reducing anxiety in dental patients through VR imagery relaxation programs [24]. Therefore, VR technology is a useful educational and therapeutic tool, as it helps reconstruct an individual’s knowledge or cognitive structure through interaction with the subject [19,25], and it is effective in inducing a high sense of immersion by providing realistic information [23]. In addition, education using VR technology induces interaction with the subject, enables experiential learning with a high level of realism, and serves as an educational method that supports self-directed learning. It is applied in combination with information delivery and relaxation techniques [15,19,26]. Therefore, in this study, a professional approach is required to reflect the unique characteristics of high-risk pregnant women in preterm labor and to enhance their pregnancy health care practices. The Cox Subject Health Behavior Interaction Model is an appropriate nursing intervention framework for this purpose. Based on the Interaction Model of Client Health Behavior (IMCHB), we developed a prenatal education program using VR for hospitalized pregnant women in preterm labor and verified its effectiveness.

### Purpose of the Study

This study aimed to develop, implement, and evaluate the effects of a prenatal educational program using VR technology for pregnant women hospitalized with preterm labor.

## Methods

### Research Design

This quasi-experimental study used a pre- and posttest design with a nonequivalent control group. The pretest, experimental treatment, and posttest for the experimental group were conducted after the pretest and posttest of the control group, ensuring that participants in the 2 groups did not come into contact. This design was chosen to minimize the influence of subjective outcome measurements.

### Setting and Sample

The sample size was determined based on a previous study involving education for women experiencing stress and anxiety related to preterm labor [27]. Using G* Power 3.1.9.2 (Heinrich-Heine-University), the required sample size was calculated with a significance level of .05, power 0.80, and effect size of 0.97 [28]. The appropriate sample size for both the experimental and control groups was 14, as indicated by previous findings [27]. To account for a potential 20% dropout rate, 17 participants were included in each group. A total of 34 pregnant women hospitalized for preterm labor at 2 women’s hospitals were recruited through convenience sampling; 17 participants were assigned to the control group and 17 to the experimental group. To reduce bias and enable blinding, participants were recruited from women’s hospitals with similar bed capacities. One hospital was assigned to conduct the experimental intervention, while the other was used to collect control group data. The author distributed questionnaires to participants before and after the prenatal educational program. All participants completed the preintervention questionnaire; however, 2 participants in the experimental group were excluded (1 was transferred to another hospital and 1 reported discomfort using the head-mounted display [HMD] equipment). In the control group, 1 participant was excluded due to insincere responses. Thus, data from 31 participants (experimental group, n=15; control group, n=16) were included in the final analysis.

### Ethical Considerations

The Institutional Review Board of Keimyung University approved this study (IRB No. 40525‐202007-BR-036‐01). As the study was not a randomized controlled trial and participants were not randomly assigned to intervention or control groups, it was not registered as a clinical trial. After being informed about the study’s purpose, methods, intervention details, and survey content, participants voluntarily provided written informed consent before participation. Confidentiality was ensured, and participants were informed that they could withdraw from the study at any time without negative consequences. Participants were also advised to rest as needed and withdraw from the VR training program if they experienced discomfort or dizziness during the intervention. Although no financial compensation was provided, participants were informed that the VR educational content could be beneficial for understanding preterm labor and promoting health. Author SAP consented to the use of her likeness in this paper.

### Measurements

A structured questionnaire was used to collect data on general and obstetric characteristics, state anxiety, stress related to preterm labor, uterine contractions, cervical length, pregnancy health care practice behavior, and self-efficacy (Multimedia Appendix 1).

#### State Anxiety

State anxiety was measured using the State-Trait Anxiety Inventory (STAI) [29], translated into Korean [30]. The STAI includes 20 items rated on a 4-point Likert scale, ranging from 1 (not at all) to 4 (very high). The total score ranges from 20 to 80, with higher scores indicating greater anxiety. The Cronbach α for the STAI in Kim and Shin’s [30] Korean version was 0.92, and it was also 0.92 in this study.

#### Stress of Preterm Labor

Stress related to preterm labor was assessed using an instrument developed by Kim [31]. This tool consists of 17 items rated on a 5-point Likert scale, with total scores ranging from 17 to 85. Higher scores indicate higher levels of stress. The Cronbach α value was 0.79 in Kim’s original study [31], and 0.79 in this study as well.

#### Uterine Contractions

The degree of uterine contractions was measured based on their intensity and frequency. A nonstress test tocodynamometer was used for this purpose. The electronic fetal monitoring device (Fetal Monitor Device FC 1400; designed by Bionet) was used during the experimental treatment and was already in use at the hospital where the study was conducted. Data on the intensity and frequency of uterine contractions were collected from nursing records in the medical records. To evaluate the reliability of the measurements, interobserver reliability was assessed using the intraclass correlation coefficient. In this study, the intraclass correlation coefficient was 0.80 (95% CI 0.682-0.894; *P*<.001).

#### Cervical Length

Cervical length was defined as the distance (in mm) from the internal os to the external os of the cervix, measured using a transvaginal ultrasound device [32]. The Medison Accuvix (Samsung) was used for this purpose during the experimental treatment. Data on cervical length were obtained from nursing records in the medical charts.

#### Practice Behavior of Pregnancy Health Care

The practice behavior of pregnancy health care was measured using an instrument developed by Wang and Kim [33]. The tool includes subelements such as prenatal care and education, activity and rest, medication management, physical and hygiene management, nutritional management, and mental health. The instrument consists of 17 items rated on a 5-point Likert scale, with total scores ranging from 17 to 85. Higher scores indicate greater engagement in pregnancy health care practices. The reliability (Cronbach α) reported by Wang and Kim [33] was 0.72, whereas the value in this study was 0.76.

#### Self-Efficacy in Pregnancy Health Care

Self-efficacy in pregnancy health care was measured using an instrument developed by Wang and Kim [33]. Based on the 16-question pregnancy health care self-efficacy measurement tool developed by Wang and Kim [33], the validity of the tool was verified by a panel of 6 experts. Items with a content validity index of 0.80 or higher were selected. The item “Understanding Pregnancy in the Elderly” had a content validity index of 0.16, and was therefore excluded. The final instrument consisted of 15 questions rated on a 4-point Likert scale, with the total scores ranging from 15 to 60. Higher scores indicated greater self-efficacy in pregnancy health care. Wang and Kim [33] reported a Cronbach α of 0.89, whereas in this study, the value was 0.92.

### Development of the Program

**Figure 1.**
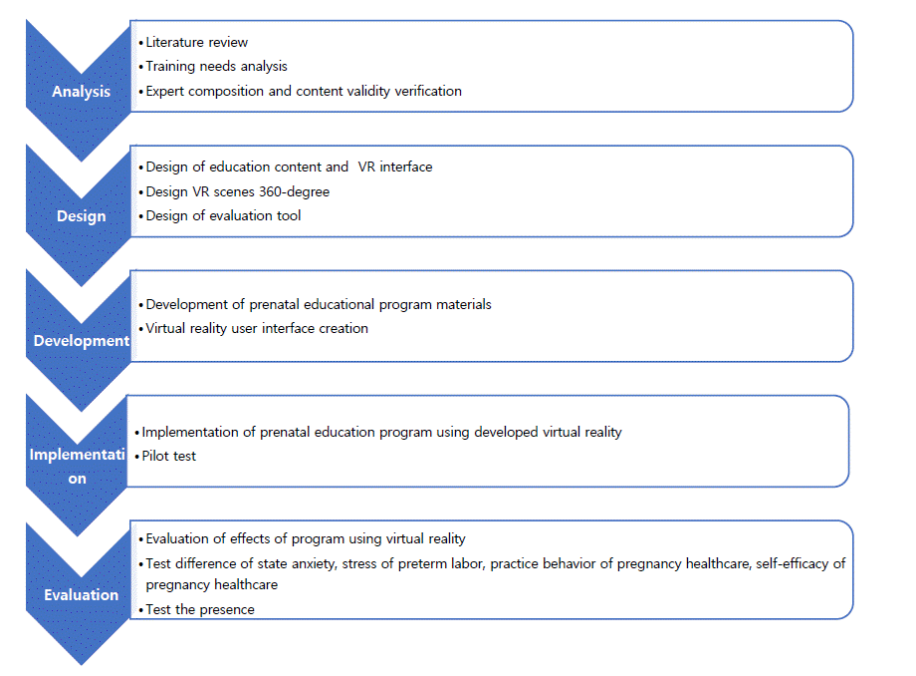
Development process of a virtual reality (VR) prenatal education program for hospitalized pregnant women with preterm labor, following the 5-step ADDIE (Analyze, Design, Develop, Implement, and Evaluate) instructional design model.

This study evaluated the effectiveness of a prenatal education program using VR for pregnant women hospitalized with preterm labor. The program was developed based on the Cox [13] IMCHB as the theoretical framework (Figure 1). To guide the development of the VR prenatal education program, the Analyze, Design, Develop, Implement, and Evaluate (ADDIE) model [34] was used. The development process followed 5 stages: analysis, design, development, implementation, and evaluation.

During the analysis step, a literature review and interviews with pregnant women hospitalized with preterm labor were conducted. The educational content included 6 subject areas with 19 detailed items: knowledge of preterm labor, relaxation, examination and treatment, symptoms and health management, nutrition and weight management, discharge education, and daily life management. The educational program was designed to incorporate the Cox IMCHB, based on the analyzed prenatal educational content (Table 1).

In the design step, the program was structured according to the subject-expert interaction elements of the Cox IMCHB: emotional support nursing, health information provision nursing, decision-making participation, and professional/technical nursing. The prenatal education program using VR was designed for 1:1 face-to-face education over 3 days. Each session followed a structured sequence: introduction (5 minutes), development (30 minutes), and conclusion (5 minutes), totaling 40 minutes. Thus, the application time of the prenatal education program for pregnant women in preterm labor during each intervention was 40 minutes.

In the introduction stage, emotional support nursing was provided through orientation, support, and encouragement to engage participants in the educational program and encourage the sharing of previous educational experiences. In the development stage, the prenatal education program using VR consisted of a situation-based content relevant to hospitalization, discharge, and daily life for pregnant women in preterm labor. Specialized content related to professional and technical nursing, including classification of labor pain, labor symptoms, symptoms of ruptured membranes, and hospital visit scenarios, was designed to help participants monitor their own condition, uterine contractions, and fetal movements. In addition, a relaxation meditation program using VR was incorporated into each session to reduce anxiety and stress in pregnant women experiencing preterm labor. Each VR-based relaxation meditation session lasted 10 minutes and included both breathing and imagery relaxation therapy, designed to provide emotional support nursing. Imagery relaxation, combined with breathing techniques and grounded in natural environments, aimed to induce deep mental comfort. The VR component enhanced this effect by providing immersive visual imagery, helping participants achieve deeper relaxation. In the final stage, the next session was introduced in advance, and emotional support, such as answering questions, offering praise, active listening, and encouragement, was repeated over the 3-day program. This approach promoted interaction and allowed pregnant women in preterm labor to independently choose and revisit information according to their needs. Throughout each intervention, the researcher continuously monitored whether participants were engaging properly in the training and whether they experienced any pain or discomfort.

**Table 1.** Structured contents of the prenatal education program using VR^a^ for hospitalized pregnant women with preterm labor, developed based on the Cox Interaction Model of Client Health Behavior.

Session number, goal, contents, and subcontents					Methods	Time (minutes)
1						40
	Understanding preterm labor and recognition of the necessity and importance of prenatal education					
		Affective support: (1) orientation; (2) emotional management (anxiety and stress management); and (3) praise and listen			VR imagery relaxation Face-to-face, phone, and message consultation	
		Provision of health information: (1) introduction to educational programs and (2) importance of pregnancy health care			VR and brochure	
		Decisional control: (1) identifying health behaviors resulting from preterm labor and (2) consultation			Pretest Face-to-face, phone, and message consultation	
		Professional or technical competencies: (1) understanding preterm labor; (2) definition and causes of preterm labor; and (3) changes in hospitalization conditions due to preterm labor			VR and brochure	
2						40
	Recognizing that physical and mental problems experienced due to preterm labor can be alleviated through VR-based prenatal education programs					
		Affective support: (1) emotional management (anxiety and stress management) and (2) praise and listen			VR imagery relaxation Face-to-face, phone, and message consultation	
		Provision of health information: (1) types, purpose, and methods of testing			VR and brochure	
		Decisional control: (1) health problems caused by preterm labor (uterine contraction or vaginal bleeding) and (2) consultation			VR and brochure Face-to-face, phone, and message consultation	
		Professional or technical competencies: (1) check uterine contractions and labor and (2) check fetal condition			VR and brochure	
3						40
	Understanding early labor reduces anxiety and stress and increases self-care performance and self-efficacy					
		Affective support: (1) emotional management (anxiety and stress management) and (2) praise and listen			VR imagery relaxation Face-to-face, phone, and message consultation	
		Provision of health information: (1) weight management and (2) bed stability management			VR and brochure	
		Decisional control: (1) health problems caused by preterm labor (labor pain, labor pain, or rupture of membranes) and (2) consultation			Pretest VR and brochure Face-to-face, phone, and message consultation	
		Professional or technical competencies: (1) daily life and discharge management (hospital visit situation); (2) check uterine contractions and labor; and (3) check fetal condition.			VR and brochure	

a VR: virtual reality.

During the development step, scenarios, flow charts, and scenes were created based on educational content validated by an expert panel consisting of obstetricians, clinical nurses, and nursing professors. The prenatal education curriculum for pregnant women hospitalized with preterm labor was structured for each session according to its goals, details, program content, educational methods, and the time required. For the VR content, filming was conducted using a 360° camera, and each scene was rendered in a 3D space. The VR educational programs were developed for use with an HMD. As relaxation meditation can be challenging to perform without guidance, the researcher (SAP) directly participated in the VR videos to lead breathing and relaxation exercises for pregnant women in preterm labor. Thus, the VR prenatal education program was developed as 2 components: an educational program and a relaxation meditation program.

**Figure 2.**
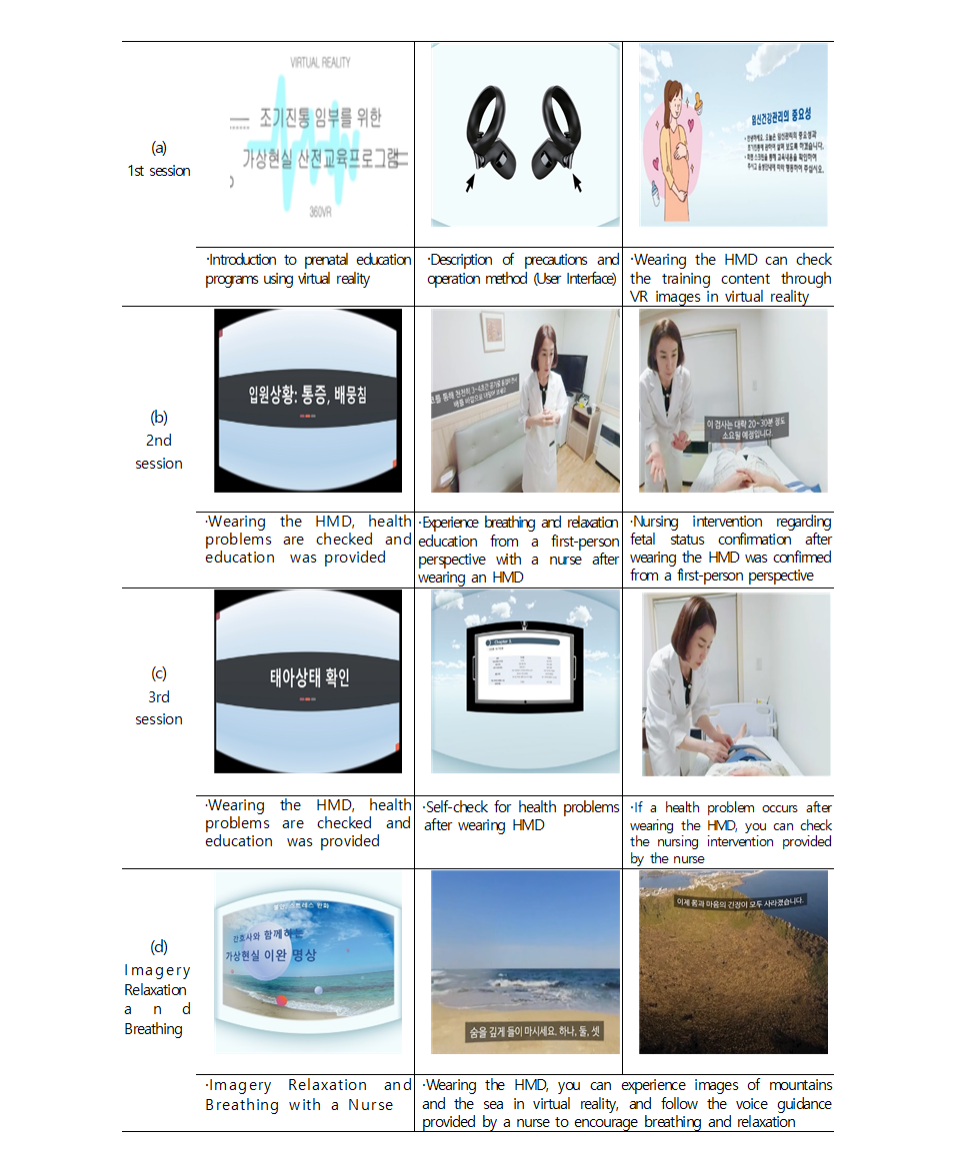
Screenshots from each session of the virtual reality (VR) prenatal education program for hospitalized pregnant women with preterm labor. (A) User interface for navigating VR content and selecting training options. (B) Educational session on uterine contractions and nursing care for abdominal tightening and pain during hospitalization. (C) Self-check session including fetal condition monitoring with feedback from a virtual nurse. (D) VR-based imagery relaxation session with a nurse, incorporating meditation and breathing exercises in a simulated natural environment. HMD: head-mounted device.

The educational VR program is titled “Virtual Reality Prenatal Education Program for Pregnant Women in Preterm Labor” and has a total running time of 20 minutes. The relaxation meditation program is titled “Virtual Reality Relaxation Meditation Program With a Nurse” and has a total running time of 10 minutes (Figure 2).

**Figure 3.**
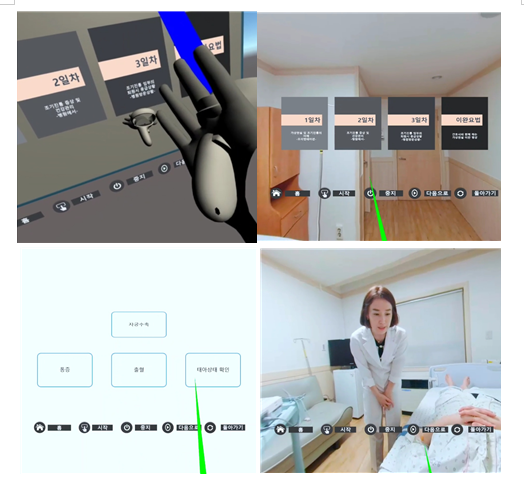
Screenshots of the user interface used in the virtual reality (VR) prenatal education program. The interface enables hospitalized pregnant women with preterm labor to navigate sessions; select educational content on topics such as pain, uterine contractions, and fetal health; and interact with a virtual nurse in a 360-degree immersive environment.

To maximize interaction in VR, a user interface was implemented that recognizes finger movements [35]. This feature allows for independent participation in anxiety-reduction and nursing education interventions. The screen configuration of the VR user interface was designed to automatically transition from one screen to the next, following a predefined flowchart. The interface included 4 main menus and 5 selection menus positioned at the bottom of the screen (Figure 3).

During the implementation step, a preliminary survey was conducted before the evaluation phase to confirm the feasibility of operating and applying the developed prenatal education program using VR in actual clinical practice. The expert group included 1 VR expert, 1 nursing professor, and 1 head nurse from a women’s specialty hospital. Based on their feedback, final modifications and enhancements were made—specifically, adjustments to the subtitle location and font size on the implementation screen, as well as the symbolic and intuitive redesign of the icon images used in the interface menu buttons.

During the evaluation step, Lee’s [36] presence evaluation tool was used to assess the level of presence experienced in the VR-based prenatal education program. The expert group gave a total mean score of 180.20 (SD 1.78) out of 190 points. In addition, a user evaluation was conducted with 3 pregnant women hospitalized for preterm labor, who rated the program with a total mean score of 181.66 (SD 10.11), indicating the program was appropriate and well-received.

### Data Collection

Data were collected before and after the prenatal education program using a self-reported, face-to-face method. The pre- and postintervention questionnaires took about 20 minutes to complete and included items on general and obstetric characteristics, state anxiety, stress related to preterm labor, practice behavior of pregnancy health care, and self-efficacy.

### Statistical Analysis

All data were analyzed using SPSS version 24 (IBM Corp). A *P* value of <.05 was considered statistically significant. General and obstetric characteristics, along with homogeneity tests, were analyzed using proportions (%) and means with SDs, applying the *χ*^2^ test, Fisher exact test, and independent, unpaired, 2-tailed *t* test. The Kolmogorov-Smirnov test was used to assess normality. The effects on state anxiety, the stress of preterm labor, uterine contractions, cervical length, practice behavior of pregnancy health care, and self-efficacy were analyzed using independent *t* tests, the Wilcoxon signed rank test, and the Mann-Whitney *U* test.

## Results

### Characteristics of the Participants

The general characteristics, obstetric characteristics, and dependent variables did not significantly differ between the experimental and control groups, indicating that the groups were homogeneous (Table 2).

**Table 2.** Baseline demographic and clinical characteristics of hospitalized pregnant women with preterm labor, compared between the experimental group (n=15) and control group (n=16).

Characteristics and category	Experimental group (n=15)	Control group (n=16)	*t* test (*df*) or chi-square (*df*)^a^	*P* value
Age (years), mean (SD)	30.26 (3.63)	31.43 (2.50)	1.50 (29)	.30
Spouse, n (%)			0.96 (29)	.34
0	0 (0)	1 (6)		
1	15 (100)	15 (94)		
Level of education, n (%)			0.10 (29)	.92
High school	4 (27)	4 (25)		
University	11 (73)	12 (75)		
Occupation, n (%)			*1.06 (29)*	.30
Yes	3 (20)	6 (38)		
No	12 (80)	10 (63)		
Gestational age (weeks), mean (SD)	30.05 (2.54)	30.94 (1.94)	1.10 (29)	.28
Number of children^b^, n (%)			*3.18 (2)*	.32^b^
0	8 (53)	7 (44)		
1	4 (27)	8 (50)		
2	3 (20)	1 (6)		
Experience of delivery, n (%)			*0.32 (1)*	.41
Yes	7 (47)	9 (56)		
No	8 (53)	7 (44)		
Method of previous delivery^c^, n (%)			*0.83 (14)*	.47
Normal	6 (86)	6 (67)		
Cesarean section	1 (14)	3 (33)		
Times of delivery^c^, n (%)			*2.37 (2)*	.32^c^
0	8 (53)	7 (44)		
1	4 (27)	8 (50)		
2	3 (20)	1 (6)		
Experience of abortion^b^, n (%)			*0.81 (1)*	.37
Yes	6 (40)	9 (56)		
No	9 (60)	7 (44)		
Times of abortion^b^^,^^d^, n (%)			*0.83 (2)*	>.99^b^
1	4 (67)	6 (67)		
2	2 (33)	2 (22)		
≥3	0 (0)	1 (11)		
Antenatal care^b^, n (%)			*0.87 (2)*	.63^b^
Not at all	2 (13)	1 (6)		
Irregularly	4 (27)	3 (19)		
Regularly	9 (60)	12 (75)		
Planned pregnancy, n (%)			*0.26 (1)*	.60
Yes	8 (53)	10 (63)		
No	7 (47)	6 (38)		
Knowledge level related to preterm labor^b^, n (%)			*0.31 (2)*	>.99^b^
Very well	0 (0)	0 (0)		
Well	0 (0)	0 (0)		
Usually	3 (20)	4 (25)		
Not know	9 (60)	8 (50)		
Not at all	3 (20)	4 (25)		
Necessity of preterm labor education^b^, n (%)			*0.08 (1)*	>.99^b^
Very necessary	12 (80)	13 (81)		
Necessary	3 (20)	3 (19)		
Moderate necessary	0 (0)	0 (0)		
Not necessary	0 (0)	0 (0)		
Not necessary at all	0 (0)	0 (0)		
Information sources^b^^,^^e^, n (%)			*0.31 (2)*	. 85^b^
Doctors and nurses	1 (7)	4 (25)		
Internet	15 (100)	15 (94)		
Antenatal classes	1 (7)	4 (25)		
Books	0 (0)	4 (25)		
State anxiety (score), mean (SD)	58.46 (9.03)	56.87 (10.31)	−0.45 (29)	.65
Stress of preterm labor (score), mean (SD)	56.80 (7.88)	57.25 (7.04)	0.16 (29)	.87
Uterine contractions, mean (SD)				
Frequency of uterine contraction (times/min)	3.16 (0.87)	3.12 (0.34)	−0.17 (29)	.87
Intensity of uterine contraction (mm Hg)	33.33 (10.63)	35.00 (16.12)	0.33 (29)	.74
Cervical length (mm), mean (SD)	30.00 (6.48)	30.25 (7.73)	0.09 (29)	.92
Practice behavior of pregnancy health care (score), mean (SD)	63.73 (6.12)	65.43 (5.22)	0.83 (29)	.41
Self-efficacy in pregnancy health care (score), mean (SD)	36.66 (5.30)	35.81 (4.83)	−0.46 (29)	.64

a Chi-square test (*df*) values are in italics.

b Fisher exact test.

c 16 respondents with delivery experience.

d 15 respondents with abortion experience (“yes” respondents: 6 in the experimental group and 9 in the control group).

e Information sources in multiple responses.

### Effects of the Program

The state anxiety (*t*_28.72_=2.80, *P*=.009) and stress of preterm labor (*t*_26.85_=3.34, *P*=.002) scores after the prenatal education program using VR were significantly lower in the experimental group than in the control group. Similarly, the frequency of uterine contractions (*t*_28.93_=3.08, *P*=.004) and intensity of uterine contractions (*t*_29_=4.75, *P*<.001) were significantly reduced in the experimental group compared with the control group. Furthermore, the degree of change in cervical length was significantly greater in the experimental group than in the control group (*U*=54.50, *P*=.009). The experimental group also showed significantly higher scores in practice behavior of pregnancy health care (*t*_28.48_=−2.07, *P*=.047) and self-efficacy in pregnancy health care (*t*_24.57_=−4.12, *P*<.001) than the control group (Table 3).

**Table 3.** Differences in state anxiety, stress of preterm labor, uterine contractions, cervical length, practice behavior, and self-efficacy in pregnancy health care treatment between the 2 groups (N=31).

Variables	Experimental group (n=15), mean (SD)	Control group (n=16), mean (SD)	*t* test (*df*)/*U* test^a^	*P* value
State anxiety (score)	44.53 (7.58)	52.93 (8.96)	2.80 (28.72)	.009
Stress of preterm labor (score)	48.66 (4.82)	55.87 (6.91)	3.34 (26.85)	.002
Frequency of uterine contraction (times/min)	1.00 (0.75)	1.84 (0.76)	3.08 (28.93)	.004
Intensity of uterine contraction (mm Hg)	10.13 (3.97)	23.43 (10.11)	4.75 (29)	<.001
Cervical length (mm)	33.93 (5.33)	31.62 (7.38)	*54.50*	.009
Practice behavior of pregnancy health care (score)	71.60 (5.86)	67.37 (5.47)	−2.07 (28.48)	.047
Self-efficacy in pregnancy health care (score)	46.93 (2.93)	40.81 (4.98)	−4.12 (24.57)	.001

a *U* test value is in italics.

## Discussion

### Principal Findings

This program was based on the Cox (2003) IMCHB as the theoretical foundation. In addition, we developed and evaluated the effects of a prenatal educational program using VR, guided by the ADDIE model, for hospitalized pregnant women in preterm labor. The program was developed through analysis, design, development, implementation, and evaluation.

Nurses must provide professional and specific knowledge and information about preterm labor to help pregnant women navigate this critical period [37,38]. Therefore, this program is significant in that it reflects the educational needs of pregnant women in preterm labor, uses a VR program as an intervention strategy within the expert/technical interaction domain, and adopts an individualized intervention approach involving experts.

In the first step of program analysis, the educational program was designed based on the Cox IMCHB, incorporating the elements of emotional support, health information, decision-making participation, and professional nursing. It addressed the needs of hospitalized women with preterm labor across 6 main topics. The VR-based 1-on-1 education appeared to enhance engagement and interaction between experts and participants, which may have contributed to reduced anxiety and improved health behaviors among hospitalized pregnant women.

In the design step, the overall educational content was structured based on the findings from the analysis phase. During the development of the VR-based educational program, an educational content scenario was drafted, and corresponding VR scenes were composed and designed. To enable pregnant women hospitalized with preterm labor to access information efficiently, a user interface using hand gestures was developed. The educational content was organized consistently with the analysis phase to enhance participants’ understanding of the program.

In the development step, the VR prenatal education program was created to support self-guided relaxation therapy through immersive, first-person experiences using an HMD. Real voice narration, natural soundscapes, and imagery of mountains and the sea were incorporated to enhance emotional engagement and promote stress relief. The background for the relaxation meditation therapy using actual footage of mountains and sea, as imagery relaxation in a natural environment is known to support voluntary cognitive focus, reduce stress, and induce a sense of comfort [39,40].

In the implementation step, the program was revised and refined by experts to ensure its feasibility for application in clinical settings. Captions were added at the bottom of the screen to improve comprehension and highlight key information. Footage of nurses providing care was included to help reduce viewer anxiety. The final video was formatted for viewing with an HMD capable of displaying 360° video. The gesture-based user interface allowed for interactive engagement without requiring users to memorize specific hand movements.

In the evaluation step, the VR prenatal education program was tested with 15 participants in the experimental group and 16 in the control group. The results demonstrated that the experimental group experienced significantly lower levels of anxiety, preterm labor stress, and uterine contractions, as well as increased cervical length, improved pregnancy health care behaviors, and greater self-efficacy compared with the control group. These findings confirm that the prenatal education program using VR effectively reduced state anxiety among hospitalized pregnant women in preterm labor.

This study builds on previous research in which anxiety in pregnant women with preterm labor was measured following the application of an educational program emphasizing interaction elements between participants and experts, based on the Cox Subject Health Behavior Interaction Model. In that study, the average anxiety score decreased from 46.44 before the intervention to 38.89 after the intervention. Similar findings have been reported in other studies involving pregnant women with preterm labor [15]. Hospitalized pregnant women in preterm labor often experience high levels of anxiety due to uncertainty about their symptoms and prognosis, the risk of recurrent labor, concerns about fetal health, and the unpredictability of pregnancy outcomes [8].

Such anxiety can negatively affect both the mother and the fetus, making anxiety-reducing interventions essential. Emotion-focused coping strategies, particularly when supported by tailored information and education, are more effective than problem-focused approaches. This study suggests that combining education with relaxation therapy, tailored to the unique needs of these women, may reduce anxiety—possibly by activating the parasympathetic nervous system.

Following the application of the VR prenatal education program, stress related to preterm labor was significantly reduced in the experimental group. This finding aligns with previous studies indicating that VR featuring natural scenes, when combined with techniques such as breathing therapy, effectively promotes mental relaxation and reduces stress [22,41]. These results underscore the potential for future programs using VR-based relaxation in natural environments to positively impact both physiological and emotional well-being.

After implementing the prenatal education program using VR, the degree of uterine contractions in the experimental group was significantly reduced compared with the control group. In a previous study, Park and Sung [42] assessed the effect of music therapy on uterine contraction frequency in pregnant women with preterm labor. Their results showed a decrease in the average frequency of uterine contractions in the experimental group by 0.35 points, from 3.00 before intervention to 2.65 after. In the control group, contraction frequency decreased by 0.33 points, from 2.78 to 2.44. Although this indicated a tendency for relaxation therapy through music to reduce uterine muscle activity, the findings were not statistically significant—unlike the results of our study. These findings are supported by previous research indicating that imagery-based interventions and abdominal breathing can help relax uterine muscles and reduce the need for pain relief in women with preterm labor [43,44].

In addition, prior studies have shown that changes in cervical length vary based on individual characteristics of women with preterm labor [45]. Psychological interventions, such as relaxation therapy combined with drug treatment, have been associated with significant increases in cervical length and reductions in uterine contractions [46]. Given that symptoms of preterm labor often go unrecognized by patients [47], and many report difficulty identifying contractions along with a need for clearer education [48,49], this VR-based prenatal program presents a promising intervention. By combining education, guided imagery, and breathing relaxation, the program may contribute to a reduced risk of premature labor and improved cervical stability. These findings suggest that ongoing, multifaceted nursing interventions—tailored to individual symptoms—have the potential to positively influence physiological outcomes in pregnant women with preterm labor.

After applying the VR prenatal care program, the experimental group showed significantly higher self-efficacy in pregnancy health management compared with the control group. This finding is consistent with previous studies that used mobile or expert-led prenatal education programs, which enhanced self-efficacy by providing specific information and encouraging self-directed learning [33,50]. Accurate, tailored education combined with interactive relaxation sessions helped boost participants’ confidence and intrinsic motivation, leading to improved practice behavior and more active engagement [33,51].

Pregnant women hospitalized with preterm labor reported that the VR prenatal education program made pain management strategies easy to understand and remember, thereby enhancing their coping ability. The immersive, experience-based format appeared to increase engagement, self-management, and internal motivation by incorporating expert interaction and professional information. These findings suggest that the program may be a promising option as a customized nursing intervention for future clinical applications.

### Limitations

This study had several limitations. Blinding was not feasible due to the nature of the intervention, which may have introduced bias—particularly in the measurement of subjective outcomes such as anxiety and stress—despite efforts to ensure fair and consistent delivery. To reduce the risk of contamination and minimize potential bias, the experimental and control groups were assigned to 2 different women’s hospitals before the intervention. This site-level allocation helped maintain separation between groups; however, potential differences in hospital care may have influenced the outcomes. Furthermore, the generalizability of the findings is limited, as the study was conducted in only 2 hospitals and involved a convenience sample of pregnant women hospitalized with preterm labor. Future studies should be conducted in more diverse settings and populations to replicate and extend these results. Importantly, the study design was strengthened by the use of pre- and postintervention assessments and validated measurement instruments. Although the wireless HMD used in the intervention provided mobility, it presented technical challenges such as focus adjustment and user comfort, which should be considered when applying such tools in future clinical settings. While the quasi-experimental design and educational nature of the intervention were unlikely to affect participant safety or rights, this limitation does not compromise the validity of the findings. Despite these constraints, the program effectively reduced anxiety and stress in pregnant women with preterm labor. To enhance practical utility in clinical practice, future educational interventions should adopt flexible, context-specific approaches that reflect varying levels of clinical risk and complexity, rather than relying on a uniform regulatory framework.

### Conclusion

Based on the Cox IMCHB, this study developed a VR prenatal education program tailored to the complex needs of women with preterm labor, providing individualized support across physical, emotional, educational, and professional domains. The program’s effectiveness was validated using both objective and subjective tools, enhancing its reliability. The positive outcomes suggest its potential to inform the development of future prenatal education protocols. As science and technology continue to advance, the importance of interdisciplinary and convergent research becomes increasingly evident. This study is meaningful in its integration of VR technology into nursing education, and future research should explore multidisciplinary approaches aligned with evolving care methods.

## Supplementary material

10.2196/75585 Multimedia Appendix 1 Questionnaires used to assess anxiety, stress, self-efficacy, and pregnancy health care behaviors.

## Abbreviations

ADDIE: Analyze, Design, Develop, Implement, and Evaluate
HMD: head-mounted display
IMCHB: Interaction Model of Client Health Behavior
STAI: State-Trait Anxiety Inventory
VR: virtual reality
