# Supplementary material for: Development and Effect of Prenatal Education Programs Using Virtual Reality for Pregnant Women Hospitalized With Preterm Labor: Experimental Study
Source: J Med Internet Res. 2025 Jun 30;27:e75585. doi: 10.2196/75585 (PMC12234401; doi:10.2196/75585)
Supplement: Multimedia Appendix 1 [file jmir-v27-e75585-s001.docx]

**[Research questionnaire]**

**General Characteristics**

**Please check the appropriate box for each question.**

1. Age (Year)? (______)

2. Do you have a spouse?

□ 1) Yes □ 2) NO □ 3) Other

3. Religion?

□ 1) Christianity □ 2) Buddhism □ 3) Catholic □ 4) None □ 5) Other

4. Level of Education?

□ 1) equal to or more than university □ 2) less than university □ 3) Other

5. Occupation?

□ 1) teacher □ 2) specialized job □ 3) office worker

□ 4) sales □ 5) self-employment □ 6) production job

□ 7) housewife □ 8) student □ 9) Other

6. Cohabiting family?

□ 1) alone □ 2) spouse

□ 3) spouse, child □ 4) parents

□ 5) Other

**Obstetric characteristics**

**Please check the appropriate box for each question.**

1. gestational age now? (____Weeks____Days)

2. pregnancy planned?

□ 1) Yes □ 2) No

3. Have you ever experienced childbirth?

□ 1) Yes □ 2) No

4. Kind of Delivery? (If question 3 is "yes")

□ 1) Normal vaginal delivery (how many times ＿＿) □ 2) C-sec (how many times＿＿)

5. How many children?

□ 1) 1 person □ 2) 2 person □ 3) 3 person □ 4) none

6. Have you ever had an abortion?

□ 1) Yes (how many times ＿＿) □ 2) No

7. Have you ever been diagnosed with and treated for preterm?

□ 1) Yes □ 2) No

8. Have you received prenatal care?

□ 1) Not at all □ 2) Irregularly □ 3) Regularly

9. How much do you know about health problems related to preterm labor?

□ 1) Very well □ 2) Well □ 3) Usually □ 4) Not known □ 5) Not at all

10. Do you think education about health issues related to preterm labor is necessary?

□ 1) Very necessary □ 2) Necessary □ 3) Moderate □ 4) Not necessary □ 5) Not necessary at all

11. What methods have you used to obtain knowledge and information about health issues related to preterm labor?

(Please check all)

□ 1) medical staff (Nurse, Doctor) □ 2) Internet □ 3) □ 4) Antenatal classes □ 5) Books □ 6) Other

**State-Trait Anxiety (State)**

The following questions are used by people to express themselves.

Check the box that best describes your current feeling.

*Reverse questionnaire Items

| Questionnaire Items | | Not at all | Somewhat | Moderate | Very high |
| --- | --- | --- | --- | --- | --- |
| 1* | I feel calm |  |  |  |  |
| 2* | I feel secure |  |  |  |  |
| 3 | I am tense |  |  |  |  |
| 4 | I feel strained |  |  |  |  |
| 5* | I feel at ease |  |  |  |  |
| 6 | I feel upset |  |  |  |  |
| 7 | I am presently worrying over possible misfortunes |  |  |  |  |
| 8* | I feel satisfied |  |  |  |  |
| 9 | I feel frightened |  |  |  |  |
| 10* | I feel comfortable |  |  |  |  |
| 11* | I feel self-confident |  |  |  |  |
| 12 | I feel nervous |  |  |  |  |
| 13 | I am jittery |  |  |  |  |
| 14 | I feel indecisive |  |  |  |  |
| 15* | I am relaxed |  |  |  |  |
| 16* | I feel content |  |  |  |  |
| 17 | I am worried |  |  |  |  |
| 18 | I feel confused |  |  |  |  |
| 19* | I feel steady |  |  |  |  |
| 20* | I feel pleasant |  |  |  |  |

**Stress of preterm labor**

The following questions are designed to determine the level of stress of preterm labor

Please check the appropriate box for each question.

| Questionnaire Items | | Not at all | Somewhat | Moderate | Considerably | Extremely |
| --- | --- | --- | --- | --- | --- | --- |
| 1 | I'm bored staying in the hospital and not being able to do anything. |  |  |  |  |  |
| 2 | I'm worried that preterm labor will come back |  |  |  |  |  |
| 3 | I am worried that the baby will be born prematurely and end up in an incubator. |  |  |  |  |  |
| 4 | I find it uncomfortable to use a toilet bowl to urinate in bed. |  |  |  |  |  |
| 5 | I am uncomfortable because I cannot wash my hair or take a bath due to absolute rest. |  |  |  |  |  |
| 6 | I am worried that amniotic fluid may leak or bleed before delivery. |  |  |  |  |  |
| 7 | I feel uncomfortable because I can't do the leisure activities I want. |  |  |  |  |  |
| 8 | I'm worried that I won't be able to take care of my housework and family due to my hospitalization. |  |  |  |  |  |
| 9 | I feel upset because I feel like the fetus is suffering because of me. |  |  |  |  |  |
| 10 | I am concerned that uterine contractions may have a negative effect on the fetus. |  |  |  |  |  |
| 11 | I feel uncomfortable having to get help from people around me when doing anything. |  |  |  |  |  |
| 12 | I feel uncomfortable due to the inability to attain sufficient sleep while hospitalized. |  |  |  |  |  |
| 13 | I am ashamed of my frequent vaginal examinations. |  |  |  |  |  |
| 14 | I experience upset feelings due to my inability to fully feel the happiness of pregnancy like others. |  |  |  |  |  |
| 15 | I have concerns about whether I will be able to sufficiently cover the cost of hospitalization. |  |  |  |  |  |
| 16 | I am worried that constipation may cause premature birth. |  |  |  |  |  |
| 17 | I'm worried that my husband will love the baby less. |  |  |  |  |  |

**Practice behavior of pregnancy healthcare**

The following questions are designed to determine the level of practice behavior of pregnancy healthcare

Please check the appropriate box for each question.

| Questionnaire Items | | Not at all | Almost | Sometimes | Good | Very Good |
| --- | --- | --- | --- | --- | --- | --- |
| 1 | I don't smoke. |  |  |  |  |  |
| 2 | I don't drink alcohol. |  |  |  |  |  |
| 3 | I take medicine after consulting a doctor. |  |  |  |  |  |
| 4 | I keep my body clean. |  |  |  |  |  |
| 5 | I wear low heeled shoes. |  |  |  |  |  |
| 6 | I weigh myself regularly. |  |  |  |  |  |
| 7 | I exercise regularly. |  |  |  |  |  |
| 8 | I look for pregnancy and childbirth information. |  |  |  |  |  |
| 9 | I receive regular prenatal checkups at the hospital. |  |  |  |  |  |
| 10 | I don't lift heavy things. |  |  |  |  |  |
| 11 | I get enough rest. |  |  |  |  |  |
| 12 | I avoid excessive sex life. |  |  |  |  |  |
| 13 | I eat food that is not salty. |  |  |  |  |  |
| 14 | I reduce my intake of bread or carbonated drinks. |  |  |  |  |  |
| 15 | I eat three meals a day regularly. |  |  |  |  |  |
| 16 | I try to have a happy heart. |  |  |  |  |  |
| 17 | I engage in prenatal care that suits my needs. |  |  |  |  |  |

**Self-efficacy of pregnancy healthcare**

The following questions are designed to determine the level of practice behavior of pregnancy healthcare

Please check the appropriate box for each question.

| Questionnaire Items | | Not at all | Somewhat | Moderate | Very high |
| --- | --- | --- | --- | --- | --- |
| 1 | I know the purpose of the tests I receive at the hospital. |  |  |  |  |
| 2 | I can manage normal body changes during pregnancy. |  |  |  |  |
| 3 | I know the importance of fetal movement. |  |  |  |  |
| 4 | I can cope appropriately if I have vaginal bleeding. |  |  |  |  |
| 5 | I can cope appropriately with lower abdominal pain. |  |  |  |  |
| 6 | I know the symptoms of gestational hypertension and can deal with it (manage it). |  |  |  |  |
| 7 | I know the symptoms of preterm labor and can deal with them (manage them). |  |  |  |  |
| 8 | I can be careful about my daily life to avoid giving preterm |  |  |  |  |
| 9 | I am confident that I can manage my weight gain during pregnancy to within 15 kg. |  |  |  |  |
| 10 | I know the importance of taking iron pills during pregnancy. |  |  |  |  |
| 11 | I know the importance of diet during pregnancy. |  |  |  |  |
| 12 | I know situations when I shouldn't exercise. |  |  |  |  |
| 13 | I am confident in controlling my sex life during pregnancy. |  |  |  |  |
| 14 | I can take good care of my teeth during pregnancy. |  |  |  |  |
| 15 | I can manage to stress well during pregnancy. |  |  |  |  |

Thank you for your answers.
